# Intelligent Fault Diagnosis System for Running Gear of High-Speed Trains

**DOI:** 10.3390/s25175269

**Published:** 2025-08-24

**Authors:** Shuai Yang, Guoliang Gao, Ziyang Wang, Shengfeng Zeng, Yikai Ouyang, Guanglei Zhang

**Affiliations:** 1 School of Electrical and Electronic Engineering, Shijiazhuang Tiedao University, Shijiazhuang 050043, China; yang_s@stdu.edu.cn (S.Y.); a2328182263@163.com (G.G.); 15938509301@163.com (Z.W.); 17736664872@163.com (S.Z.); ku3991@163.com (Y.O.); 2 National Engineering Research Center for Colloidal Materials, Shandong University, Jinan 250100, China

**Keywords:** rail transit, fault diagnosis of train running gear, TimesNet, embedded systems

## Abstract

Conventional rail transit train running gear fault diagnosis mainly depends on routine maintenance inspections and manual judgment. However, these approaches lack robustness under complex operational environments and elevated noise levels, rendering them inadequate for real-time performance and the rigorous accuracy standards demanded by modern rail transit systems. Furthermore, many existing deep learning–based methods suffer from inherent limitations in feature extraction or incur prohibitive computational costs when processing multivariate time series data. This study represents one of the early efforts to introduce the TimesNet time series modeling framework into the domain of fault diagnosis for rail transit train running gear. By utilizing an innovative multi-period decomposition strategy and a mechanism for reshaping one-dimensional data into two-dimensional tensors, the framework enables advanced temporal-spatial representation of time series data. Algorithm validation is performed on both the high-speed train running gear bearing fault dataset and the multi-mode fault diagnosis datasets of gearbox under variable working conditions. The TimesNet model exhibits outstanding diagnostic performance on both datasets, achieving a diagnostic accuracy of 91.7% on the high-speed train bearing fault dataset. Embedded deployment experiments demonstrate that single-sample inference is completed within 70.3 ± 5.8 ms, thereby satisfying the real-time monitoring requirement (<100 ms) with a 100% success rate over 50 consecutive tests. The two-dimensional reshaping approach inherent to TimesNet markedly enhances the capacity of the model to capture intrinsic periodic structures within multivariate time series data, presenting a novel paradigm for the intelligent fault diagnosis of complex mechanical systems in train running gears. The integrated human–machine interaction system includes a comprehensive closed-loop process encompassing detection, diagnosis, and decision-making, thereby laying a robust foundation for the continued development of train running gear predictive maintenance technologies.

## 1. Introduction

The train running gear constitutes the critical load-bearing and motion guidance system of railway vehicles. It primarily comprises wheelsets, axle boxes, suspension systems (including primary and secondary suspensions), braking devices, and bogie frames. The running gear serves three essential functions, which are supporting and transmitting various loads between the vehicle body and track infrastructure, guiding the vehicle for safe and stable operation along the railway alignment, and mitigating impacts and vibrations during train operation to ensure ride comfort and system reliability [1]. Traditional rail transit train running gear fault diagnosis mainly depends on scheduled maintenance inspections and experiential manual evaluations. This approach is not only inefficient and costly but also highly susceptible to human error, thereby failing to satisfy the real-time performance and rigorous accuracy standards of modern rail transit systems [2]. With the continuous progress of sensor technology and signal processing techniques, fault diagnosis methodologies based on vibration signal analysis have gradually become a significant research focus in the academic realm [3]. Conventional signal processing techniques, including Fast Fourier Transform (FFT) [4], wavelet transform, Empirical Mode Decomposition (EMD), and so on [5], can effectively extract fault features under specific conditions. However, their robustness diminishes significantly in complex operating environments characterized by high noise levels. Moreover, these methods are ill-equipped to address intricate fault patterns involving multivariate coupling [6].

Recent developments in deep learning have introduced transformative prospects for rail transit fault diagnosis [7]. Deep learning approaches have garnered wide recognition for their superior feature extraction and classification capabilities, but advanced methods need to be developed. Convolutional Neural Network (CNN)–based methods convert one-dimensional vibration signals into two-dimensional time-frequency images, leveraging CNNs’ spatial feature extraction strengths for fault classification [8]. Nevertheless, this methodology faces inherent constraints: the trade-off between time and frequency resolution during transformation impedes precise detection of transient fault features; while two-dimensional convolutions excel at capturing spatial patterns, they inadequately model long-term temporal dependencies in time series data; additionally, the necessity to predefine window function parameters imposes artificial priors, potentially limiting adaptability to complex and dynamic fault modes. Recurrent Neural Networks (RNN) and their variant, Long Short-Term Memory (LSTM) networks, proficiently capture long-term temporal dependencies but falter when processing multivariate time series due to vanishing gradients and reduced training efficiency [9,10]. Transformer-based architectures such as Informer demonstrate robust performance in modeling long sequences. However, their self-attention mechanisms possess quadratic computational complexity, resulting in substantial processing overhead and impeding real-time, low-latency applications in rail transit scenarios [11]. To overcome these limitations, researchers have developed increasingly sophisticated models. For example, the Time-Frequency Self-Similarity Enhancement Network (TFSSEN) and its application in the fault analysis of wind turbine generators [12] demonstrate superior performance in characterizing non-stationary signals with closely spaced and overlapping frequencies. TFSSEN improves the energy concentration of time-frequency representation (TFR) through adaptive time-frequency mapping and cascaded non-local attention residual groups, achieving an average fault recognition accuracy of over 98% under variable speed and load conditions. Similarly, the Adaptive Threshold and Attention-based Tree-inspired Network (ATCATN), designed for bearing health monitoring of aero-engines under strong noise environments [13], can accurately and progressively identify fault locations and magnitudes even in the presence of significant noise interference, maintaining an accuracy rate of over 93%. These advancements illustrate the potential of integrating advanced attention mechanisms, adaptive thresholds, and hierarchical decision-making structures to address the challenges of industrial fault diagnosis in varying and noisy operational conditions. However, their application to rail transit systems—particularly for analyzing multivariable, highly periodic, and impact-rich vibration signals from operational devices, complex cross-condition scenarios, and strict real-time processing constraints—remains largely unexplored.

Several issues still persist in the domain of rail transit fault diagnosis. The choice of feature extraction methodologies is contentious. Advocates of traditional signal processing emphasize the interpretability and reliability rooted in physical principles, whereas proponents of deep learning highlight the automation and flexibility of end-to-end learning frameworks [14]. A trade-off exists between model complexity and real-time operability; academia generally endorses complex models for superior diagnostic accuracy, whereas industry prioritizes models’ deployment efficiency and responsiveness [15]. Opinions diverge on the necessity of multi-modal data fusion. Some researchers argue that single-modal vibration data suffice for effective diagnosis [16], while others stress that integrating multi-dimensional sensor modalities—including vibration, acoustic, and thermal data—substantially enhances diagnostic precision [17]. These ongoing debates underscore the necessity for fault diagnosis approaches that can flexibly balance interpretability, accuracy, real-time performance, and multi-source data integration to meet both academic research and practical engineering demands.

While TimesNet (Temporal 2D-Variation Modeling for General Time Series Analysis) introduces a novel and efficient framework for time series analysis, its effectiveness and deployment in complex industrial fault diagnosis scenarios remain to be systematically validated. Recently, the TimesNet time series modeling framework was proposed by Wu et al., which presents a novel approach to addressing these challenges [18]. By introducing a multi-period decomposition and two-dimensional spatio-temporal feature modeling architecture, TimesNet effectively transcends limitations inherent in traditional time series models. Its core innovation reshapes one-dimensional time series into two-dimensional tensor representations, enabling simultaneous modeling of intra-period temporal variations and inter-period frequency-domain evolution. Compared to conventional methods, TimesNet exhibits striking advances across diverse time series prediction tasks, achieving average performance improvements exceeding 15% [19]. Crucially, its two-dimensional convolutional architecture entails significantly lower computational complexity than Transformer models, facilitating real-time application feasibility. Nonetheless, the practical utility of TimesNet in industrial fault diagnosis, especially within rail transit, demands systematic verification. The intricacies of rail transit systems, multivariate data interdependencies, and stringent real-time diagnostic requirements pose challenges to deployment. Moreover, the transition of TimesNet from research to engineering practice, model deployment on resource-constrained embedded platforms, and the construction of integrated fault diagnosis systems also need to be investigated.

Based on the above analysis, this study presents one of the pioneering applications of the TimesNet framework in multi-class fault diagnosis for rail transit running gear. To address the multi-scale periodic characteristics of operational condition signals from high-speed train running gear, an intelligent fault diagnosis method based on the TimesNet framework is proposed. The method achieves accurate modeling under complex working conditions through one-dimensional to two-dimensional time series reconstruction combined with a multi-scale convolution mechanism. A human–machine interaction system is designed and implemented, integrating sensor signal acquisition, intelligent diagnosis, visual analysis, and maintenance decision suggestions into a closed-loop workflow, thereby validating the method’s applicability in real-world operation and maintenance scenarios. Furthermore, to meet practical engineering requirements, the model is first trained on a high-performance workstation and then transferred and deployed on the Rockchip (RK) 3588 Advanced RISC Machine (ARM)-based edge embedded platform for rigorous validation. The feature modeling capability of TimesNet in industrial multi-sensor time series data processing is both theoretically analyzed and experimentally verified, demonstrating its advantages in capturing periodic patterns and suppressing noise.

## 2. Materials and Methods

### 2.1. Dataset

#### 2.1.1. High-Speed Train Running Gear Bearing Fault Dataset

The high-speed train running gear bearing fault dataset provides authoritative experimental support for research into the intelligent diagnosis of bearing faults under complex operational conditions. Supplied by China Academy of Railway Sciences Corporation Limited [20], the dataset comprises fault test data for three critical bearing components within the CR400BF Electric Multiple Unit (EMU)—axle box bearings, gearbox bearings, and motor bearings—and encompasses fundamental fault types, including inner ring faults, outer ring faults, cage faults, and rolling element faults. The experimental protocols are meticulously designed to encompass nearly one hundred diverse operating conditions, varying by rotational speed, static load, and excitation, thereby ensuring the broad representativeness and comprehensiveness of the dataset across a wide spectrum of working scenarios.

**Figure 1:**
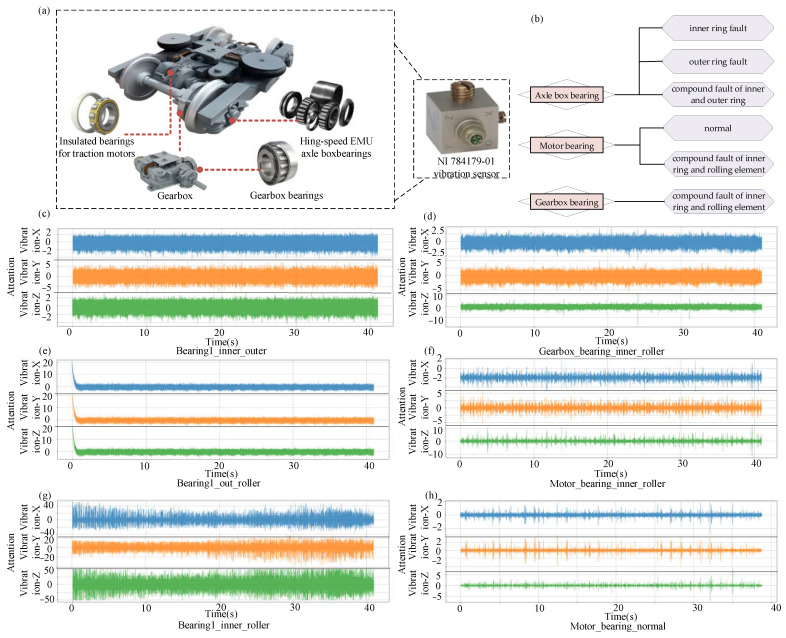
The bearing fault dataset for high-speed train running gear. (**a**) Schematic of the running gear structure and the NI 784179-01 vibration sensor; (**b**) types of fault samples; (**c**) visualization of the “Bearing1_inner_outer” fault sample data; (**d**) visualization of the “Gearbox_bearing_inner_roller” fault sample data; (**e**) visualization of the “Bearing1_out_roller” fault sample data; (**f**) visualization of the “Motor_bearing_inner_roller” fault sample data; (**g**) visualization of the “Bearing1_inner_roller” fault sample data; (**h**) visualization of the “Motor_bearing_normal” fault sample data.

For data acquisition, professional-grade National Instruments (NI) vibration sensors were utilized to ensure both reliability and consistency of the measured signals. As depicted in Figure 1a, vibration monitoring signals were captured using the NI 784179-01 sensor (National Instruments Corporation, Austin, TX, USA), featuring a sensitivity of 100 mV/g and a sampling rate of 25.6 kHz. This setup enables the simultaneous acquisition of vibration characteristics in the axial (X), radial (Y), and vertical (Z) directions. For each fault type under the stipulated working conditions, data were collected over intervals of approximately two minutes, providing an abundant volume of samples for subsequent time series feature analysis.

A comprehensive classification system is established within the dataset, distinguishing between single and compound fault categories. Specifically, the axle box bearing samples encompass three fault types: inner ring fault, outer ring fault, and compound fault of the inner and outer rings. The motor bearing samples include two types, normal and compound fault of the inner ring and rolling element, while the gearbox bearing samples contain a single class, compound fault of the inner ring and rolling element. Representative vibration signal patterns of each fault type are presented in Figure 1b–h. This multi-tiered classification system establishes a robust foundation for evaluating the multi-class recognition capabilities of the TimesNet model.

#### 2.1.2. Multi-Mode Fault Diagnosis Datasets of Gearbox Under Variable Working Conditions

To further enhance the fault coverage of gearbox components across a wide range of operating conditions and to address the paucity of gearbox-related fault data in the previously referenced high-speed train running gear bearing fault dataset, the “multi-mode fault diagnosis datasets of gearbox under variable working conditions” is adopted as a critical supplement [21]. This dataset was co-collected by the Shanghai MCC5 Group and Tsinghua University, utilizing a two-stage parallel gearbox test rig as the experimental platform. Comparative analysis with multiple benchmark datasets, as summarized in Table 1, highlights the dataset’s distinctive strengths in terms of fault type diversity and the complexity of operating scenarios. The integration of this dataset provides essential data support for the development of a unified fault diagnosis model that encompasses various components of the running gear.

**Figure 2:**
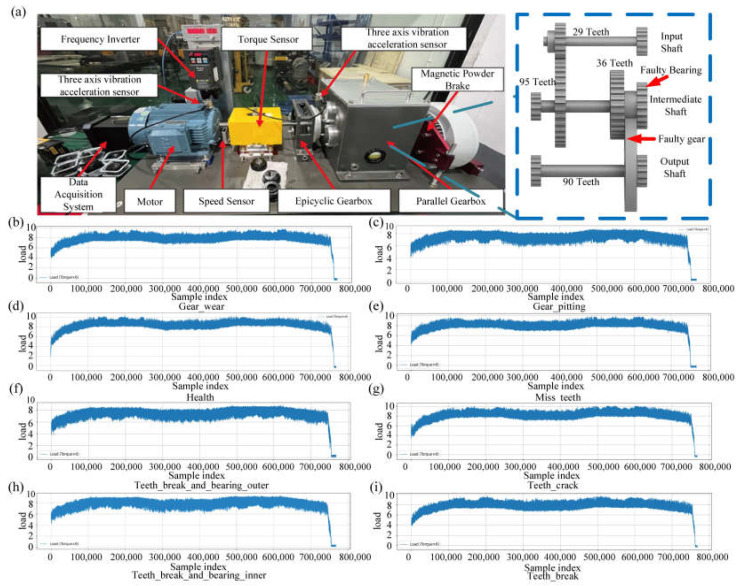
Multimodal gearbox fault diagnosis dataset. (**a**) Schematic illustration of the experimental platform and gearbox configuration; (**b**) visualization of the “Gear Wear” fault sample data; (**c**) visualization of the “Gear Pitting” fault sample data; (**d**) visualization of the “Healthy” state sample data; (**e**) visualization of the “Missing Teeth” fault sample data; (**f**) visualization of the “Teeth Breakage Combined with Outer Race Bearing Fault” sample data; (**g**) visualization of the “Teeth Crack” fault sample data; (**h**) visualization of the “Teeth Breakage Combined with Inner Race Bearing Fault” sample data; (**i**) visualization of the “Teeth Breakage” fault sample data.

The multimodal gearbox fault diagnosis dataset employed in this study was collected using a high-precision data acquisition system. As illustrated in Figure 2a, the comprehensive experimental platform consists of a three-phase asynchronous motor, a two-stage parallel gearbox, a magnetic powder brake, a torque sensor (Model S2001, Zhonghang Electronic Measuring Instruments Co., Ltd., Xinxiang, China, with an accuracy of ±0.5% F.S.), and two sets of three-axis accelerometers (TES001V, Beijing Test Equipment Co., Ltd., Beijing, China, with a sensitivity of 100 mV/g and a sampling frequency of 12.8 kHz). These accelerometers are strategically positioned at the motor drive end and the intermediate shaft of the gearbox, enabling the acquisition of multichannel and multivariable vibration signals. Additionally, the system supports synchronous recording of torque and speed signals, thereby ensuring the comprehensive capture of all critical information. The vibration signals are configured along the X, Y, and Z axes, establishing a solid basis for multidimensional feature extraction and modeling.

To accommodate a breadth of operating conditions and diverse fault scenarios, the dataset comprises an extensive collection of diagnostic samples. It consists of eight distinct fault types, including single gear faults and compound faults involving both gear and bearing components. These encompass classic fault states such as normal operation, missing tooth, gear wear, gear pitting, gear cracking, and tooth breakage, as well as compound conditions like tooth breakage combined with inner-race bearing faults and tooth breakage combined with outer-race bearing faults. Each fault category is further differentiated by varying degrees of severity. The experimental parameters span a range of rotational speeds (1000, 2000, and 3000 rpm) and loads (10 and 20 Nm), with a comprehensive matrix of steady-state and dynamic loading configurations to ensure the representativeness of multiple operating conditions. Each raw data segment is recorded over a 60 s interval, yielding a total of 240 samples. Visual representations of signals corresponding to various fault types are shown in Figure 2b–i. This dataset significantly enriches the diversity of fault morphologies and operating modes specific to gearbox components and effectively complements the high-speed train running gear bearing fault dataset, thereby providing a robust data foundation for the evaluation of intelligent fault diagnosis systems for both high-speed train running gears.

### 2.2. TimesNet

**Figure 3:**
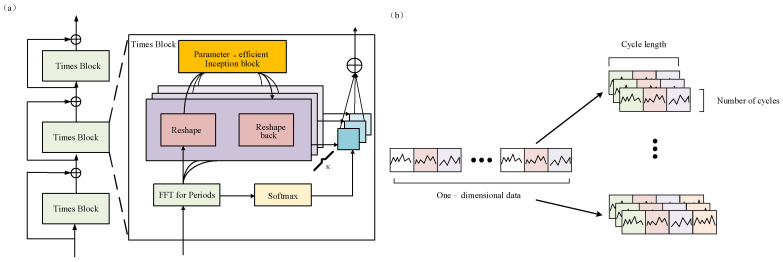
TimesNet. (**a**) Overview of the TimesNet architecture; (**b**) schematic depiction of the transformation from one-dimensional to two-dimensional representation.

To overcome the inherent limitations of conventional one-dimensional modeling approaches in multivariate time series analysis, this study adopts the TimesNet framework as the core algorithm for fault diagnosis. The principal innovation of this approach lies in extending complex temporal variations from a one-dimensional time axis to a two-dimensional spatial representation. Through multi-period decomposition, the framework thoroughly excavates the latent patterns embedded within time series data. As illustrated in the Figure 3, the overall architecture of TimesNet comprises multiple stacked TimesBlocks [25]. Each TimesBlock incorporates essential components, including FFT-based period estimation, two-dimensional reshaping, a parameter-efficient Inception-based feature extraction module, and adaptive fusion. This constitutes a complete processing pipeline traversing from one-dimensional time series to two-dimensional features and subsequently reverting to one-dimensional representations. Such a design empowers the model to capitalize fully on the multi-period characteristics intrinsic to time series data, thereby providing an effective modeling paradigm for intricate multivariate time series analysis. TimesNet initiates the process by conducting frequency-domain analysis on the input one-dimensional time series using the Fast Fourier Transform (FFT) to identify dominant periodic components. Specifically, for an input sequence $X_{1D}\in R^{T\times C}$, the model estimates the k most significant period lengths $\left\{p_{1},\cdots ,p_{k}\right\}$, and their corresponding frequencies $\left\{f_{1},f_{2},\cdots ,f_{k}\right\}$, via the Period() function. Leveraging this period information, the original one-dimensional sequence is reshaped into k two-dimensional tensors:

(1) $$X_{2D}^{i}={Reshape}_{p_{i},f_{i}}\left(Padding(X_{lD})\right),i\in \{1,\cdots ,k\},$$

where $X_{2D}^{i}\in R^{p_{i}\times f_{i}\times C}$ denotes the i-th reshaped time series based on frequency $f_{i}$. The columns and rows of the tensor, respectively, capture intra-periodic and inter-periodic variations, mapped to the period length $p_{i}$. This transformation preserves multi-scale periodic features within a two-dimensional spatial structure, effectively capturing both intra- and inter-periodic dynamics.

For the reshaped two-dimensional tensors, TimesNet utilizes a parameter-efficient Inception module for feature extraction. Equipped with multi-scale two-dimensional convolutional kernels, this module is capable of simultaneously extracting local temporal dependencies within periods and long-range evolution patterns across periods:

(2) $$\begin{array}{cc} A^{l-1},\{f_{1},\cdots ,f_{k}\},\{p_{1},\cdots , & p_{k}\}=Period(X_{1D}^{l-1}),\\ & X_{2D}^{l,i}={Reshape}_{p_{i},f_{i}}(Padding(X_{1D}^{l-1})),i\in \{1,\cdots ,k\},\\ & {\hat{X}}_{2D}^{l,i}=Inception(X_{2D}^{l,i}),i\in \{1,\cdots ,k\},\\ & {\hat{X}}_{1D}^{l,i}=Trunc({Reshape}_{1,(p_{i}\times f_{i})}({\hat{X}}_{2D}^{l,i})),i\in \{1,\cdots ,k\}, \end{array}$$

where $X_{2D}^{l,i}\in R^{p_{i}\times f_{i}\times d_{model}}$ represents the *i*-th two-dimensional tensor, and Trunc() truncates padding to the original sequence length. The two-dimensional convolutions within the Inception module efficiently aggregate both local correlations among adjacent time points (column-wise) and period-to-period dependencies (row-wise), thus enabling the effective modeling of cross-time-scale relationships in multivariate time series. To optimize parameter efficiency, all *k* two-dimensional tensors share the Inception module, rendering model complexity independent of the hyperparameter *k*. Following two-dimensional convolution, feature representations are reverted to a one-dimensional format.

Finally, the model adaptively fuses outputs based on the amplitude weights of each periodic component:

(3) $$\begin{array}{c} {\hat{A}}_{f_{1}}^{l-1},\cdots ,{\hat{A}}_{f_{k}}^{l-1}\begin{array}{cc} & =Softmax\left(A_{f_{1}}^{l-1},\cdots ,A_{f_{k}}^{l-1}\right) \end{array}\\ X_{lD}^{l}=\sum_{i=1}^{k}{\hat{A}}_{f_{i}}^{l-1}\times {\hat{X}}_{lD}^{l,i}. \end{array}$$

The amplitude ***A****_i_* reflects the relative significance of each frequency and period, signifying the importance of each transformed two-dimensional tensor and enabling the model to integrate features from various periods in a differentiated manner.

During the operation of a high-speed train’s running gear, multi-modal vibration signals generated by key components such as gears and bearings under varying working conditions typically exhibit the following characteristics:

Strong periodicity: The signals are dominated by rotational and meshing frequencies, showing prominent fundamental frequency and harmonic components in the frequency domain.

Working condition dependence: Variations in load and speed cause shifts in energy distribution and amplitude changes.

Significant impact components: Local structural damage introduces short-duration, high-amplitude transient impact waves.

Multi-channel correlation: Signals from different sensors (e.g., radial, axial, and vertical directions) exhibit both shared and distinct time-frequency patterns.

At the core of the TimesNet model lies a one-dimensional to two-dimensional time series reconstruction strategy. This approach maps one-dimensional long sequences into two-dimensional tensors based on the signal’s periodic structure, enabling two-dimensional convolution operations to simultaneously model intra-period variations and inter-period trends. The multi-scale convolutional kernels (based on the Inception architecture) further enhance the model’s frequency-domain coverage, allowing it to capture both dominant harmonic components and damage-specific frequency bands under varying rotational speeds and load conditions. Additionally, the multi-head attention mechanism adaptively assigns weights across channels, highlighting the contribution of key component vibration signals to the overall feature representation. Residual connections and normalization strategies improve the stability and robustness of long-sequence modeling in the presence of noise.

Therefore, from the perspective of signal characteristics and modeling mechanism alignment, the structural design of TimesNet is theoretically well-suited for effective feature extraction and pattern recognition of the multi-modal, highly periodic, and impact-rich vibration signals from high-speed train running gear. This provides a solid modeling foundation for high-precision fault diagnosis.

## 3. Experimental Results

To evaluate the function of the TimesNet model in fault diagnosis for high-speed train running gears, this study established a multifaceted experimental framework encompassing model training, ablation studies, comparative experiments, the human–machine interaction system, and engineering deployment. The experiments utilized both the high-speed train running gear bearing fault dataset and the multimodal gearbox fault diagnosis dataset under varied operating conditions. Data was divided into training, validation, and test sets according to a 6:2:2 ratio. During the training process of the TimesNet model, the main parameters are set as follows: the number of input channels of the encoder (Enc In) is 3 (indicating the dimension of input features), the input sequence length of the decoder (Dec In) is 7 (representing the number of time steps, unit: steps), the number of output categories (C Out) is 7 (dimensionless), the model feature dimension (d_model) is 64 (dimensionless), the number of heads in multi-head attention (n_heads) is 8 (number of heads), the number of encoder layers (e_layers) is 2 (number of layers), the number of decoder layers (d_layer) is 1 (number of layers), the hidden dimension of the feedforward fully connected layer (d_FF) is 128 (number of neurons), the total number of training epochs (Train Epochs) is 30 (iterations), the batch size (Batch Size) is 32 (samples per batch), and the initial learning rate (Learning Rate) is 0.001 (dimensionless). These parameter settings ensure the scientific rigor and reproducibility of the experimental results. This multidimensional experimental scheme provides a robust foundation for the comprehensive assessment of the TimesNet model’s performance in complex time series fault diagnosis tasks.

### 3.1. Model Training

#### 3.1.1. Training Results of the High-Speed Train Running Gear Bearing Fault Dataset

**Figure 4:**
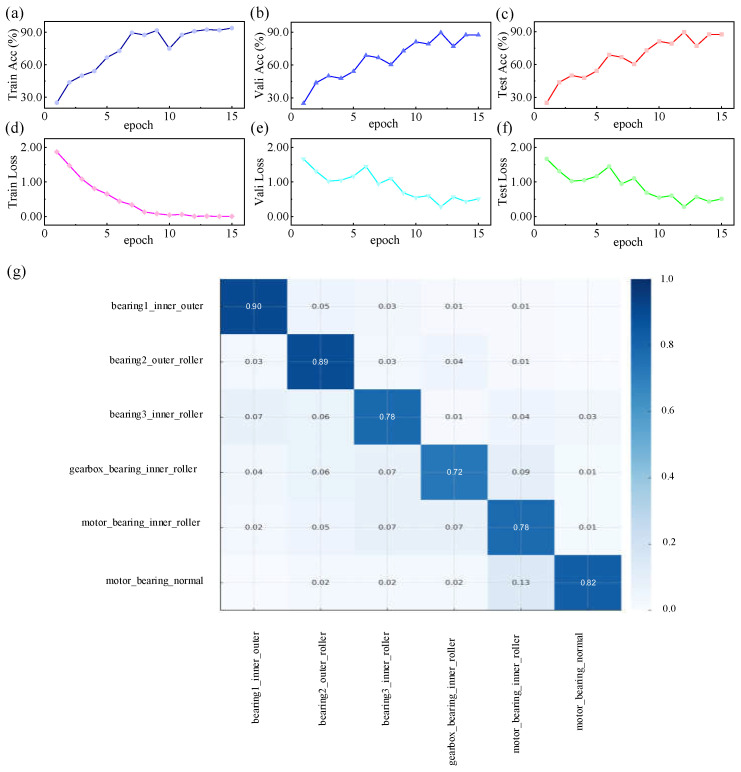
Training outcomes for the high-speed train running gear bearing fault dataset. (**a**) Training set accuracy versus number of training epochs; (**b**) validation set accuracy versus number of training epochs; (**c**) test set accuracy versus number of training epochs; (**d**) training set loss function versus number of training epochs; (**e**) validation set loss function versus number of training epochs; (**f**) test set loss function versus number of training epochs; (**g**) normalized confusion matrix.

During the training phase of the high-speed train running gear bearing fault dataset, key model performance indicators such as accuracy and loss steadily improved and eventually stabilized as the number of training epochs increased. The trajectories of these metrics exhibited a clear optimization trend, suggesting that the model possesses stable convergence and robust generalization capability. Ultimately, the test set accuracy reached 0.917, demonstrating the model’s high efficiency in anomaly detection and classification. Further analysis of the normalized confusion matrix revealed that the model achieved classification accuracy above 0.75 for most bearing fault categories (e.g., bearing1_inner_outer, bearing2_outer_roller, bearing3_inner_roller, motor_bearing_inner_roller, motor_bearing_normal). This indicates minimal misclassification or missed detection, highlighting the model’s strong capability in extracting discriminative features and accurately identifying fault types. For the gearbox_bearing_inner_roller category, although some degree of misclassification was observed, the recognition rate remained relatively high at 0.72. These results confirm that the model maintains strong discriminative performance even under conditions of limited sample size and noise interference, see Figure 4.

#### 3.1.2. Training Results of the Multi-Mode Fault Diagnosis Datasets of Gearbox Under Variable Working Conditions

During training on the multi-modal gearbox fault diagnosis dataset under various operating conditions, the model’s accuracy steadily increases while the loss metric rapidly decreases and gradually stabilizes as the number of training epochs progresses. This trend demonstrates that the model maintains excellent stability throughout the optimization process and is capable of effectively learning and extracting time series features, even in highly complex and multi-modal environments. The final validation results show that the test set accuracy reaches approximately 0.875, reflecting the model’s strong discriminative capability. A detailed analysis of the normalized confusion matrix reveals that classification accuracy exceeds 0.71 for the majority of fault categories, including gear_pitting, gear_wear, healthy, miss_teeth, teeth_break, teeth_break_and_bearing_inner, teeth_break_and_bearing_outer, and teeth_crack. This indicates that the TimesNet model demonstrates superior performance in accurately identifying most typical fault types and normal states. Furthermore, the confusion matrix shows that misclassifications are primarily confined to adjacent categories, with no widespread errors or omissions. These findings further confirm the TimesNet architecture’s strong discriminative power in multi-class fault classification and its overall robustness.

The confusion matrix results offer further insights into the relationship between the physical signal mechanisms of different fault types and their classification performance. Fault categories such as gear tooth breakage and surface pitting in the MMFD-VWC dataset, and severe outer/inner race defects in the HSRGBF dataset, demonstrate high recognition accuracies. From a signal mechanism perspective, these faults generate pronounced periodic impacts and significant amplification of specific harmonic components at characteristic fault frequencies, resulting in highly distinctive time–frequency patterns. When such features are input into the TimesNet model, its multi-period decomposition and two-dimensional temporal feature modeling effectively capture these localized high-intensity responses, thereby enabling accurate and consistent classification. In contrast, fault categories with subtle mechanical differences—such as healthy bearings versus those with very mild degradation, or inner race defects versus roller element defects—tend to produce vibration signals with similar medium- and high-frequency energy distributions. These similarities lead to overlapping feature representations in the learned embedding space, resulting in occasional misclassifications. This underscores the inherent challenge of distinguishing between fault modes with closely related physical mechanisms using the current signal acquisition setup.

**Figure 5:**
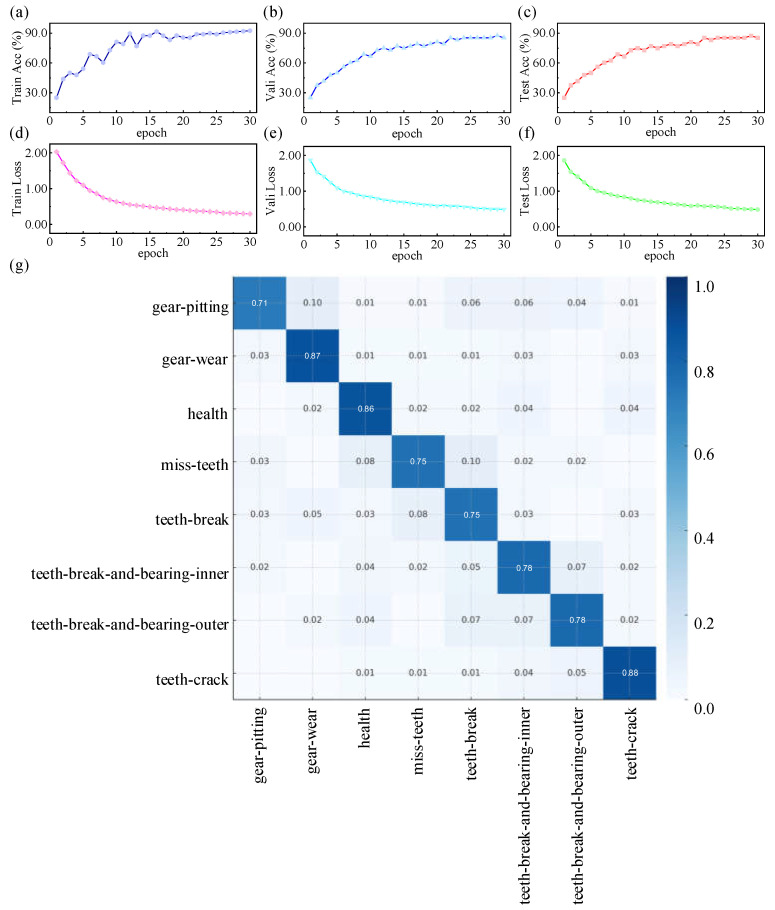
Training results of the multi-mode gearbox fault diagnosis dataset under multiple operating conditions. (**a**) Training set accuracy versus number of training epochs; (**b**) validation set accuracy versus number of training epochs; (**c**) test set accuracy versus number of training epochs; (**d**) training set loss function versus number of training epochs; (**e**) validation set loss function versus number of training epochs; (**f**) test set loss function versus number of training epochs; (**g**) normalized confusion matrix.

Overall, the experimental findings demonstrate that TimesNet achieves high-precision fault diagnosis for running gear under diverse and complex operating conditions. The model’s discriminative capacity is reflected not only in its superior overall classification accuracy but also in its consistently balanced recognition performance across a wide range of typical fault categories, see Figure 5.

### 3.2. Ablation Study

**Figure A1:**
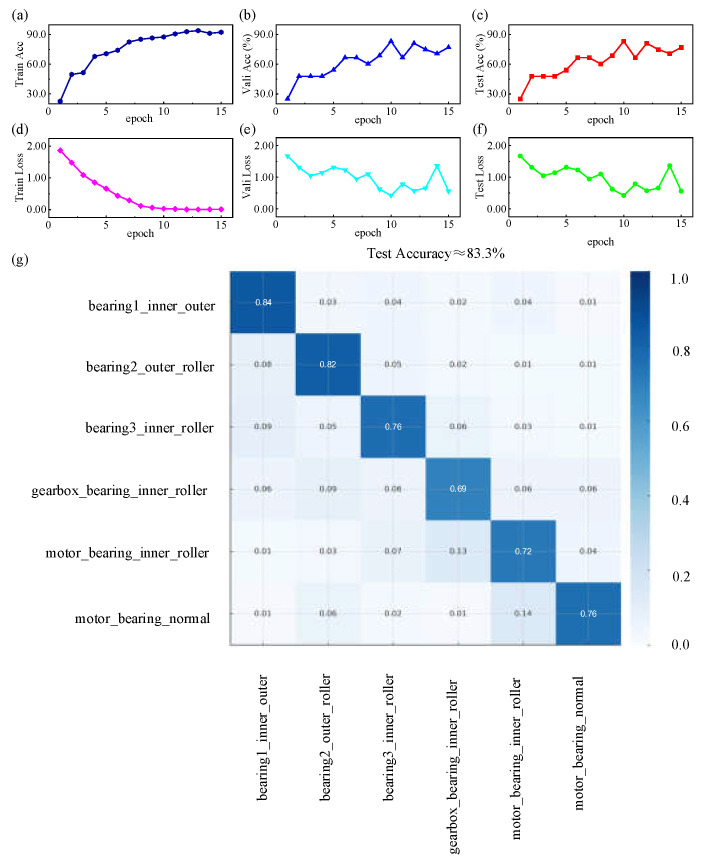
Training outcomes for the high-speed train running gear bearing fault dataset. (**a**) Training set accuracy versus number of training epochs, (**b**) validation set accuracy versus number of training epochs, (**c**) test set accuracy versus number of training epochs, (**d**) training set loss function versus number of training epochs, (**e**) validation set loss function versus number of training epochs, (**f**) test set loss function versus number of training epochs, (**g**) normalized confusion matrix.

**Figure A2:**
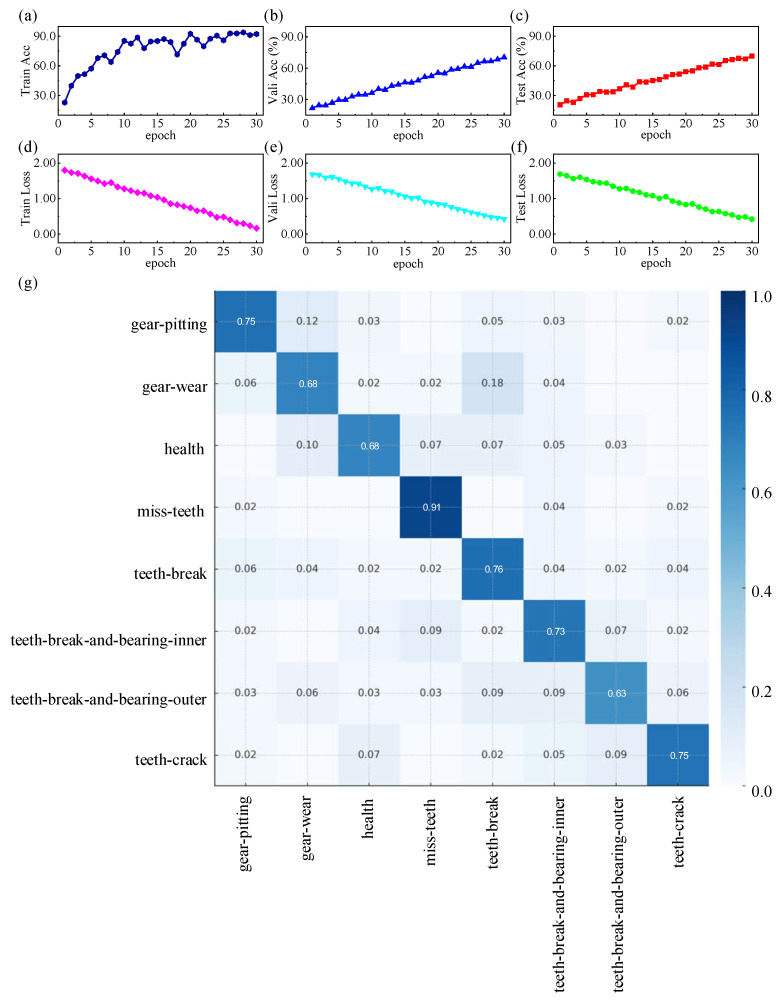
Training results of the multi-mode gearbox fault diagnosis dataset under multiple operating conditions. (**a**) Training set accuracy versus number of training epochs, (**b**) validation set accuracy versus number of training epochs, (**c**) test set accuracy versus number of training epochs, (**d**) training set loss function versus number of training epochs, (**e**) validation set loss function versus number of training epochs, (**f**) test set loss function versus number of training epochs, (**g**) normalized confusion matrix.

To systematically evaluate the practical significance of the 1D-to-2D reshaping mechanism in the TimesNet model, an ablation study was conducted. This reshaping process constitutes the core innovation of TimesNet, wherein one-dimensional time series are transformed into two-dimensional tensor structures according to their periodic characteristics. This transformation enables the model to simultaneously capture both intra-periodic and inter-periodic variations. In the control experiment, the original 1D-to-2D reshaping step in TimesNet was omitted. Instead, the one-dimensional time series was directly input into a one-dimensional convolutional network (1D Convolution) of equivalent depth for feature extraction. All other aspects of the network architecture, hyperparameters, and training protocol were maintained to ensure experimental fairness and the validity of comparative analysis. Training, validation, and test sets were each evaluated independently. Performance was assessed using multidimensional metrics, including accuracy, loss, and confusion matrices. Detailed results are provided in Appendix A of the ablation study (Figure A1 and Figure A2). Results indicated that removal of the 1D-to-2D reshaping mechanism and exclusive reliance on conventional 1D convolution led to a marked decline in model performance across all datasets. In particular, the validation and test set accuracies decreased by an average of approximately 10%, while the loss increased substantially. Comparative analysis of confusion matrices further revealed a significant rise in classification errors for certain categories within the ablated model, indicating diminished discriminative capabilities. Further examination demonstrated that the 1D-to-2D reshaping mechanism, by facilitating multi-period decomposition, produces a two-dimensional structural representation of time-series data. This empowers the model to discern both fine-grained intra-period patterns and broad inter-period trends, thereby substantially enhancing its ability to identify complex temporal structures and generalize across diverse scenarios. Without this mechanism, the model reverts to a conventional one-dimensional approach, which fails to fully exploit multi-scale periodic features, resulting in degraded performance. Overall, the ablation study conclusively demonstrates the indispensable role of the 1D-to-2D reshaping mechanism in TimesNet. This innovation significantly augments the model’s capacity to capture the intrinsic periodic structure of multivariate time series data and serves as a critical foundation for improved overall performance.

**Figure A3:**
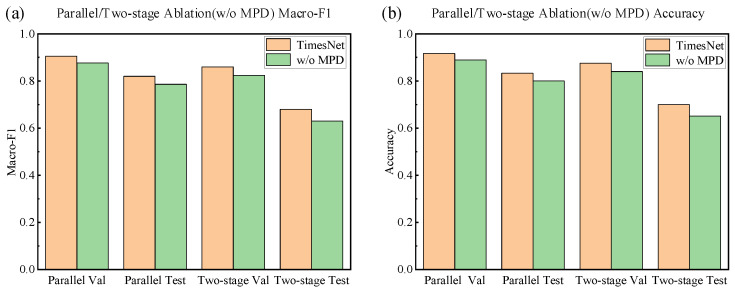
Experimental results of TimesNet and w/o MPD. Two-stage denotes the multi-mode fault diagnosis dataset for gearboxes under varying working conditions, whereas Parallel refers to the bearing fault dataset from the running gear of high-speed trains. (**a**) The macro-average F1 scores of the three models across the two datasets, (**b**) the test accuracy of the three models on both datasets.

As shown in Figure A3, to further validate the effectiveness of the multi-period decomposition (MPD) mechanism, the multi-period reconstruction process in TimesNet was removed (denoted as w/o MPD), while all other network structures and hyperparameters were kept unchanged. The original one-dimensional time series was then directly fed into the subsequent feature extraction layers for comparative experiments. The experiments were conducted on the HSRGBF and MMFD-VWC datasets, with evaluation metrics including Macro-F1 and Accuracy. As illustrated in the figure, the removal of MPD led to a performance decline across all settings to varying extents, with an average decrease of approximately 3% to 5% in Macro-F1 and approximately 3% to 4% in Accuracy. These results demonstrate that multi-period decomposition effectively captures multi-scale periodic patterns in time series, enhances the model’s discriminative capability, and serves as an essential component for TimesNet to achieve high-precision fault identification.

### 3.3. Comparison Experiment

To evaluate the effectiveness and superiority of the proposed TimesNet model for multivariate time series classification, four representative baseline networks were selected for comprehensive comparison: Informer, Long Short-Term Network (LSTNet) [26], InceptionTime: Finding AlexNet for Time Series Classification (InceptionTime) [27], and Convolutional Networks with Oriented 1D Kernels (ConvNeXt1D) [28]. All models were trained and evaluated under identical experimental settings and preprocessing protocols. Performance assessments were conducted on two datasets: the high-speed train running gear bearing fault (HSRGBF) dataset and the multi-mode gearbox fault diagnosis dataset under variable working conditions (MMFD-VWC). All experiments were carefully controlled for stochastic factors and hyperparameters to ensure reproducibility.

**Figure 6:**
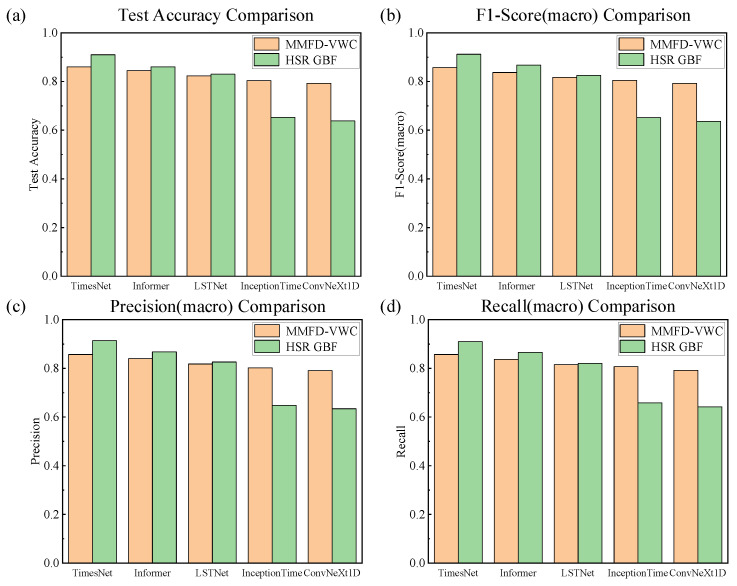
Comparative experimental results of the three models, MMFD-VWC denotes the multi-mode fault diagnosis datasets of gearbox under variable working conditions, while HSRGBF corresponds to the running gear bearing fault dataset of high-speed trains. (**a**) Test accuracies of the three models on both datasets; (**b**) macro-average precisions of the three models on both datasets; (**c**) macro-average recalls of the three models on both datasets; (**d**) macro-average F1-scores of the three models on both datasets.

For performance evaluation, a comprehensive set of mainstream classification metrics was employed, including test accuracy, macro-average precision, macro-average recall, and macro-average F1 score. These metrics provide a holistic assessment of the model’s overall discriminative capability as well as its balanced performance across diverse category distributions. As shown in Figure 6, the TimesNet model consistently outperforms the baseline models across all evaluation metrics on both datasets. A comparative analysis of the structural characteristics among different models is presented. Informer employs the ProbSparse self-attention mechanism to model long-term dependencies while improving computational efficiency. Although it surpasses traditional recurrent networks in performance, Informer has limitations in capturing complex multivariable interactions, especially under high-dimensional variable dependencies. LSTNet combines one-dimensional convolution with an LSTM structure to capture both local and global temporal dependencies. However, its dependence on convolutional and recurrent mechanisms constrains its ability to model nonlinear and complex variable relationships, leading to relatively lower and less balanced classification performance compared to Transformer-based approaches. InceptionTime achieved competitive results on certain metrics, yet exhibited instability across datasets, likely due to its reliance on deep 1D convolutional blocks for temporal feature extraction, which lack explicit modeling of cross-period dependencies. ConvNeXt1D yielded the lowest overall scores, indicating limited adaptability for multivariate fault signal classification, particularly under varying operating conditions. In contrast, TimesNet introduces a novel multi-period decomposition and two-dimensional temporal feature modeling framework, effectively addressing the limitations of the aforementioned models in capturing cross-variable and cross-scale dependencies. Consequently, TimesNet achieves both high precision and high recall across all categories and delivers optimal performance on comprehensive evaluation metrics such as the F1 score. Beyond improving overall accuracy, TimesNet ensures balanced performance across different categories, thereby demonstrating superior robustness and generalization in a wide range of diverse and hierarchical time series classification tasks.

### 3.4. Human–Machine Interaction System

**Figure 7:**
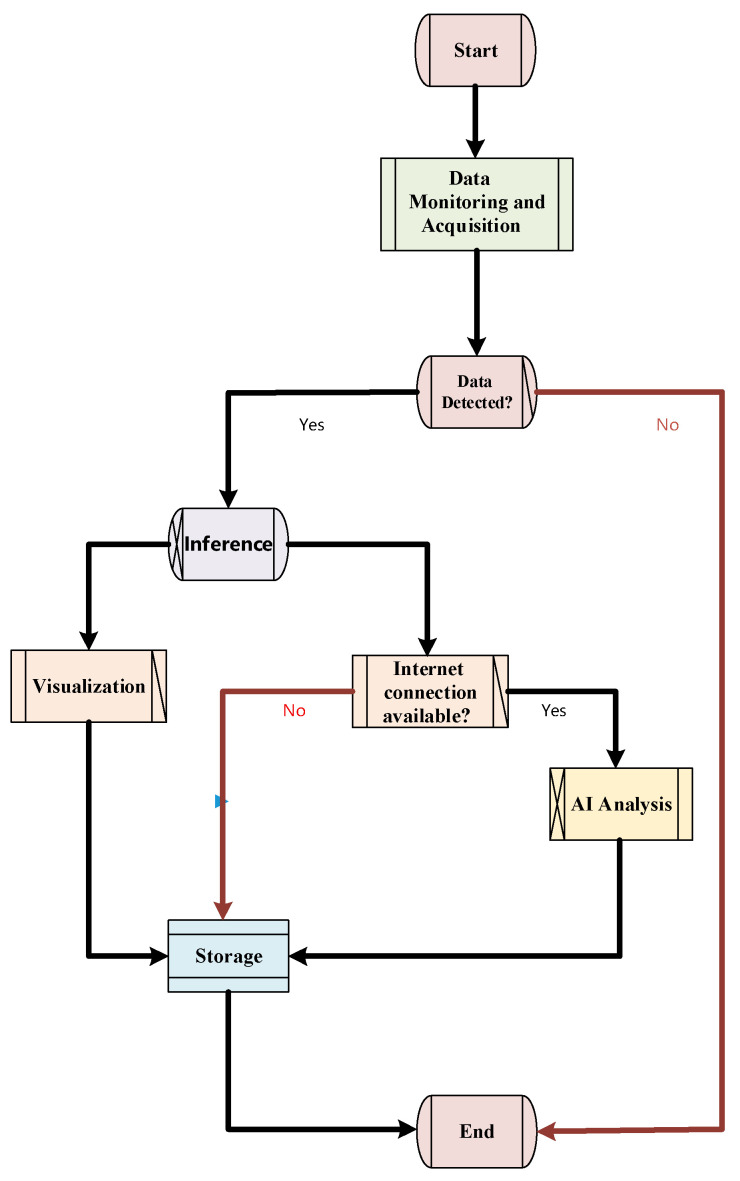
Schematic diagram illustrating the overall workflow of the human–machine interaction system.

To achieve the intelligent diagnosis of train running gear faults and enable seamless human–machine interaction, this study developed an integrated graphical diagnostic system encompassing data collection, intelligent reasoning, result visualization, Artificial Intelligence (AI)-driven analysis, and report storage. The overall workflow of the human–machine interaction system is illustrated in Figure 7. Firstly, the system automatically monitors and detects .npy and .csv directories in USB flash drives or other external storage devices through the data collection module (see detect_usb and collect_npy_files functions), thereby achieving intelligent recognition and loading of original fault samples. Once valid data is detected, the system prompts the user via the graphical interface to select samples for inference and proceeds to the subsequent processing stage. If no required data is detected, the system issues an exception alert through the Tkinter interface, halts further operations, and displays the message “No .npy/ or .csv/ directories detected”. After data validation and initiation of inference, the system enters the inference phase. Using a thread pool (ThreadPoolExecutor), it asynchronously executes the run_inference module in the background, completing the deep learning-based fault identification process. Upon completion of inference, the system automatically associates the corresponding .csv or Excel files based on the identified fault categories. Within the Matplotlib (version 3.7.1) visualization environment, the _plot_csv and _plot_excel functions display fault time series or vibration data in various formats, enabling intuitive data interpretation and real-time human–machine interaction. Meanwhile, if network connectivity is available, the ask_DeepSeek (version 3.1) function asynchronously invokes the DeepSeek-reasoner large language model API to provide targeted debugging and maintenance recommendations for the predicted fault categories; otherwise, the function remains inactive and DeepSeek cannot be called. The analysis results are dynamically displayed in real time through the ScrolledText module, supporting user decision-making. All inference outputs and AI analyses can be locally archived using modules such as save_current_results and save_analysis. The system also supports integrating Matplotlib-generated visualizations with AI-derived analysis results into a unified PNG format report and exporting the analysis text in standard formats such as txt and md for archiving and future traceability. Overall, this workflow constitutes a robust closed-loop system covering data acquisition, fault inference, visualization, AI-assisted analysis, and result storage, with all core functionalities encapsulated within dedicated class methods.

### 3.5. Verification Experiment on the Embedded Development Platform

**Figure 8:**
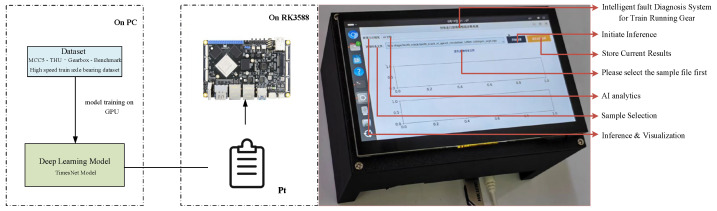
Model deployment workflow and physical device illustration.

To assess the practical feasibility of the proposed TimesNet fault diagnosis model in an embedded hardware environment, a functional verification experiment was conducted on a development board featuring ARM architecture, specifically utilizing the RK3588 processor. The LuBan Cat 5 development board served as the hardware verification platform during the inference phase. This board integrates the RK3588 octa-core processor, comprising four Cortex-A76 and four Cortex-A55 cores, achieving a maximum clock speed of 2.4 GHz and manufactured using an advanced 8-nanometer process. It is further equipped with an ARM Mali-G610 MC4 GPU and an embedded neural network processing unit (NPU) capable of 6 TOPS. The device also features a 7-inch capacitive touchscreen with a resolution of 1024 × 600, an MIPI interface, support for 5-point touch, and 24-bit RGB888 color depth. Both model training and offline inference were executed on a high-performance workstation—the Xingyun STA700—equipped with an AMD EPYC 7763 processor (64 cores), 32 GB RAM, 1 TB SSD, 16 TB enterprise-grade HDD, and an NVIDIA RTX A2000 discrete GPU. After completing training in the workstation environment, the TimesNet model was deployed directly onto the development board. Experimental results demonstrated robust functionality of the model under the ARM architecture. In this embedded context, the inference time per sample was 70.3 ± 5.8 ms (*n* = 100). The human–machine interaction interface performed reliably, accurately presenting fault diagnosis outcomes. Furthermore, during fifty consecutive inference tests, the system consistently achieved a 100% success rate, thereby ensuring diagnostic accuracy and substantiating the stability of the entire system, see Figure 8.

## 4. Discussion

This study has achieved substantial progress in both theoretical validation and engineering practice by implementing the TimesNet time-series modeling framework within the domain of rail transit fault diagnosis, thereby introducing an innovative technical solution for the intelligent diagnosis of complex mechanical systems.

However, the practical application of this research has also revealed certain technical limitations of TimesNet under specific operational scenarios, primarily in the identification of compound faults and its adaptability to extreme environments. Notably, when confronted with fault types exhibiting closely related semantic characteristics, a residual risk of misclassification remains. For instance, confusion rates between the motor_bearing_inner_roller and motor_bearing_normal categories were observed at 17% and 33%, respectively. This challenge predominantly arises from the high similarity of these states within the frequency-domain feature space. To address this, future efforts should focus on expanding the scale of training samples and refining feature engineering methodologies. Moreover, current validation efforts are predominantly based on data from a single train type. The generalizability of the model across diverse train models and varying operating conditions requires further extensive verification, presenting challenges to the widespread adoption of TimesNet in the rail transit sector. These application-related limitations delineate the key technical directions for subsequent research and development.

Future work will primarily pursue the following three directions: Firstly, multi-modal data fusion will be pivotal in enhancing diagnostic efficacy. By integrating multi-dimensional sensor information—including vibration, acoustic, temperature, and pressure data—it is anticipated that both diagnostic accuracy and environmental adaptability will be significantly improved, particularly regarding robustness in complex and high-noise operating environments. The deployment of attention mechanisms for adaptive fusion of heterogeneous sensor modalities can fully exploit their complementary strengths. Secondly, distributed collaborative diagnosis will facilitate robust fault knowledge sharing. By employing privacy-preserving technologies such as federated learning, it will be possible to share diagnostic knowledge among numerous trains and routes without compromising sensitive information, thereby constructing a comprehensive global repository of fault knowledge. This collaborative framework not only augments overall diagnostic capability but also enables the continual learning and optimization of fault prediction models. Thirdly, the advancement of edge intelligence will steer the TimesNet model toward greater efficiency and compactness. Techniques such as model compression, knowledge distillation, and neural network pruning are expected to enable intelligent diagnostic capabilities directly at the sensor level, laying a solid technical foundation for the development of an all-weather, comprehensive rail transit safety monitoring network.

## 5. Summary

This study constitutes one of the earliest efforts to apply the TimesNet time-series modeling framework to fault diagnosis in the running gear of rail transit trains. Utilizing multi-dimensional vibration sensor data, it has achieved the intelligent identification of running gear faults. Three-axis vibration signals were collected by NI 784179-01 sensors. Leveraging its multi-period decomposition mechanism, TimesNet efficiently extracts salient fault features. On high-speed train bearing and gearbox fault datasets, the model attained a diagnostic accuracy of 91.7%. Relative to conventional Informer and LSTNet approaches, this represents improvements of 7.2 and 8.7 percentage points, respectively. The system has been successfully deployed on an RK3588 embedded platform, yielding an inference time per sample of 70.3 ± 5.8 ms and achieving a 100% success rate across 50 consecutive tests, thereby satisfying the real-time monitoring requirements of rail transit applications. The developed human–machine interaction system seamlessly integrates sensor data acquisition, intelligent inference, result visualization, and AI-assisted analysis. By interfacing with the DeepSeek large-scale language model, the system enables intelligent mapping of fault types to corresponding maintenance recommendations, establishing a comprehensive closed-loop process of “sensor monitoring–fault diagnosis–maintenance decision-making.” This research substantiates the technical superiority of TimesNet in processing industrial sensor data, offering a robust train running gear fault diagnosis solution for smart operation and maintenance in rail transit.

## Figures and Tables

**Table 1:** Comparative Analysis of Representative Datasets.

	BJTU WT-Planetary Gearbox Dataset [22]	SEU Planetary Gearbox Dataset [23]	UO Variable Speed Bearing Dataset [24]	MCC5-THU Gearbox Fault Diagnosis Datasets
Number of fault types	5	5	3	7
Number of vibration signals	4	8	6	6
Sampling frequency	48 kHz	5.12 kHz	200 kHz	12.8 kHz
Sampling period	5 min	—	10 s	60 s
Key variables	Speed	Speed, Load	Speed	Speed, Load
Number of compound faults	—	—	—	2

## Data Availability

The source code utilized in this study is available upon reasonable request from the corresponding author.

## Abbreviations

The following abbreviations are used in this manuscript:

FFT	Fast Fourier Transform
EMD	Empirical Mode Decomposition
CNN	Convolutional Neural Network
RNN	Recurrent Neural Networks
LSTM	Long Short-Term Memory
TFSSEN	Time-Frequency Self-Similarity Enhancement Network
TFR	Time-Frequency Representation
ATCATN	Adaptive Threshold and Attention-Based Tree-Inspired Network
TimesNet	Temporal 2D-Variation Modeling for General Time Series Analysis
RK	Rockchip
ARM	Advanced RISC Machine
EMU	Electric Multiple Unit
NI	National Instruments
Enc In	The Number of Input Channels of the Encoder
Dec In	The Input Sequence Length of the Decoder
C Out	The Number of Output Categories
d_model	The Model Feature Dimension
n_heads	The Number of Heads in Multi-Head Attention
e_layers	The Number of Encoder Layers
d_layer	The Number of Decoder Layers
d_FF	The Hidden Dimension of the Feedforward Fully Connected Layer
MMFD-VWC	Multi-Mode Fault Diagnosis Datasets of Gearbox Under Variable Working Conditions
HSRGBF	High-Speed Train Running Gear Bearing Fault Dataset
MPD	The Multi-Period Decomposition
w/o MPD	the Multi-Period Reconstruction Process in TimesNet Was Removed
LSTNet	Long Short-Term Network
InceptionT-ime	InceptionTime: Finding AlexNet for Time Series Classification
ConvNeX-t1D	Convolutional Networks with Oriented 1D Kernels
AI	Artificial Intelligence
Two-stage	Multi-mode Fault Diagnosis Datasets of Gearbox Under Variable Working Conditions
Parallel	High-Speed Train Running Gear Bearing Fault Dataset

## References

[1] Iwnicki S., Spiryagin M., Cole C., McSweeney T. (2019). Handbook of Railway Vehicle Dynamics.

[2] Yao D., Sun Q., Yang J. (2020). Railway fastener fault diagnosis based on generative adversarial network and residual network model. Shock. Vib..

[3] Sun Y., Cao Y., Li P. (2023). Entropy feature fusion-based diagnosis for railway point machines using vibration signals based on kernel principal component analysis and support vector machine. IEEE Intell. Transp. Syst. Mag..

[4] Cooley J.W., Tukey J.W. (1965). An algorithm for the machine calculation of complex Fourier series. Math. Comput..

[5] Huang N., Shen Z., Long S. (1998). The empirical mode decomposition and the Hilbert spectrum for nonlinear and non-stationary time series analysis. Proc. R. Soc. Lond..

[6] Rico J.A., Raghavan N., Jayavelu S. Compound Fault Diagnosis for Train Transmission Systems Using Deep Learning with Fourier-enhanced Representation. Proceedings of the 2025 IEEE International Conference on Prognostics and Health Management (ICPHM).

[7] Tang Y., Zhang C., Wu J. (2024). Deep learning-based bearing fault diagnosis using a trusted multiscale quadratic attention-embedded convolutional neural network. IEEE Trans. Instrum. Meas..

[8] Galdo A.L., Guerrero-López A., Olmos P.M. (2023). Detecting train driveshaft damages using accelerometer signals and Differential Convolutional Neural Networks. Eng. Appl. Artif. Intell..

[9] Liu J., Liu F., Xie W. (2023). Track vibration sequence anomaly detection algorithm based on LSTM. Adv. Struct. Eng..

[10] Hu X., Cao Y., Tang T. (2022). Data-driven technology of fault diagnosis in railway point machines: Review and challenges. Transp. Saf. Environ..

[11] Zhou H., Zhang S., Peng J. Informer: Beyond efficient transformer for long sequence time-series forecasting. Proceedings of the AAAI Conference on Artificial Intelligence.

[12] Zhao D., Shao D., Wang T., Cui L. (2025). Time-frequency self-similarity enhancement network and its application in wind turbines fault analysis. Adv. Eng. Inform..

[13] Zhao D., Cai W., Cui L. (2024). Adaptive thresholding and coordinate attention-based tree-inspired network for aero-engine bearing health monitoring under strong noise. Adv. Eng. Inform..

[14] Li W., Wang J., Qin X. (2024). Artificial intelligence-aided detection of rail defects based on ultrasonic imaging data. Proc. Inst. Mech. Eng..

[15] Cui J., Zhong Q., Zheng S. (2022). A lightweight model for bearing fault diagnosis based on Gramian angular field and coordinate attention. Machines.

[16] Zheng Z., Song D., Xu X. (2020). A fault diagnosis method of bogie axle box bearing based on spectrum whitening demodulation. Sensors.

[17] Ye M., Yan X., Jiang D. (2024). MIFDELN: A multi-sensor information fusion deep ensemble learning network for diagnosing bearing faults in noisy scenarios. Knowl.-Based Syst..

[18] Wu H., Hu T., Liu Y. (2022). Timesnet: Temporal 2d-variation modeling for general time series analysis. arXiv.

[19] Zhang X., Yang K., Zheng L. (2024). Transformer fault diagnosis method based on timesnet and informer. Actuators.

[20] High-Speed Train Running Gear Bearing Fault Dataset. https://cstr.cn/16666.11.nbsdc.krY5GNMM.

[21] Chen S., Liu Z., He X. (2024). Multi-mode fault diagnosis datasets of gearbox under variable working conditions. Data Brief.

[22] Liu D., Cui L., Cheng W. (2023). A review on deep learning in planetary gearbox health state recognition: Methods, applications, and dataset publication. Meas. Sci. Technol..

[23] Shao S., McAleer S., Yan R. (2018). Highly accurate machine fault diagnosis using deep transfer learning. IEEE Trans. Ind. Inform..

[24] Huang H., Baddour N. (2018). Bearing vibration data collected under time-varying rotational speed conditions. Data Brief.

[25] Diederik P., Kingma, Jimmy B. (2014). A method for stochastic optimization. arXiv.

[26] Lai G., Chang W.C., Yang Y. Modeling long-and short-term temporal patterns with deep neural networks. Proceedings of the 41st International ACM SIGIR Conference on Research & Development in Information Retrieval.

[27] Fawaz H., Lucas B., Forestier G. (2020). InceptionTime: Finding AlexNet for Time Series Classification.

[28] Kirchmeyer A., Deng J. Convolutional Networks with Oriented 1D Kernels. Proceedings of the Computer Vision and Pattern Recognition.

